# Reducing Glycosphingolipid Content in Adipose Tissue of Obese Mice Restores Insulin Sensitivity, Adipogenesis and Reduces Inflammation

**DOI:** 10.1371/journal.pone.0004723

**Published:** 2009-03-23

**Authors:** Marco van Eijk, Jan Aten, Nora Bijl, Roelof Ottenhoff, Cindy P. A. A. van Roomen, Peter F. Dubbelhuis, Ingar Seeman, Karen Ghauharali-van der Vlugt, Hermen S. Overkleeft, Cynthia Arbeeny, Albert K. Groen, Johannes M. F. G. Aerts

**Affiliations:** 1 Department of Medical Biochemistry, Academic Medical Center, University of Amsterdam, Amsterdam, The Netherlands; 2 Department of Pathology, Academic Medical Center, University of Amsterdam, Amsterdam, The Netherlands; 3 Department of Organic Chemistry, Gorleaus Institute, University of Leiden, Leiden, The Netherlands; 4 Genzyme Corporation, Framingham, Massachusetts, United States of America; Mayo Clinic College of Medicine, United States of America

## Abstract

Adipose tissue is a critical mediator in obesity-induced insulin resistance. Previously we have demonstrated that pharmacological lowering of glycosphingolipids and subsequently GM3 by using the iminosugar AMP-DNM, strikingly improves glycemic control. Here we studied the effects of AMP-DNM on adipose tissue function and inflammation in detail to provide an explanation for the observed improved glucose homeostasis. Leptin-deficient obese (Lep*^Ob^*) mice were fed AMP-DNM and its effects on insulin signalling, adipogenesis and inflammation were monitored in fat tissue. We show that reduction of glycosphingolipid biosynthesis in adipose tissue of Lep*^Ob^* mice restores insulin signalling in isolated *ex vivo* insulin-stimulated adipocytes. We observed improved adipogenesis as the number of larger adipocytes was reduced and expression of genes like peroxisome proliferator-activated receptor (PPAR) γ, insulin responsive glucose transporter (GLUT)-4 and adipsin increased. In addition, we found that adiponectin gene expression and protein were increased by AMP-DNM. As a consequence of this improved function of fat tissue we observed less inflammation, which was characterized by reduced numbers of adipose tissue macrophages (crown-like structures) and reduced levels of the macrophage chemo attractants monocyte-chemoattractant protein-1 (Mcp-1/Ccl2) and osteopontin (OPN). In conclusion, pharmacological lowering of glycosphingolipids by inhibition of glucosylceramide biosynthesis improves adipocyte function and as a consequence reduces inflammation in adipose tissue of obese animals.

## Introduction

Adipose tissue essentially contributes to the obesity-driven insulin resistance syndrome as it can buffer excess of energy and secretes adipokines, which control metabolic homeostasis. However, it is not exactly understood how obesity causes this insulin resistance. Some extreme obese individuals are still able to handle increased glucose loads, whereas mildly obese individuals show severe insulin resistance and type 2 diabetes. This suggests that not the absolute amount of adipose tissue per se determines the development of insulin resistance, but that alternative explanations are possible. As long as adipose tissue can expand, in other words can store excess free fatty acids (FFA), this prevents occurrence of insulin resistance [1]–[6].

Two nonexclusive mechanisms have been proposed, which may explain how adiposity contributes to insulin resistance. It has been suggested that an increase in adiposity causes a state of low grade chronic inflammation. Endothelial cells, adipocytes and recruited inflammatory adipose tissue macrophages (ATM) all contribute to the pro-inflammatory environment in adipose tissue of obese individuals. The presence of this ensemble is thought to promote insulin resistance [7]–[14]. The initiation of the inflammatory response is incompletely understood, but is in part attributed to adipocytes. It is thought that at the onset of the inflammatory response adipocytes undergo necrotic-like death. This results in local autonomous inflammation, and the presence of ATM, forming so-called crown-like structures [15]. The recruited inflammatory macrophages produce additional chemokines and cytokines, resulting in worsening of inflammation and subsequent insulin resistance. For example, monocyte-chemo attractant protein-1 (Mcp-1, also referred to as Chemokine (C-C motif) ligand 2, Ccl-2) and its receptor, chemokine-receptor-2 (Ccr-2), have both been reported to modulate infiltration of macrophages into adipose tissue, thereby contributing to insulin resistance [16]–[18]. In addition, it has been demonstrated that osteopontin (OPN) is increased in adipose tissue of mice receiving a high fat diet. ATM are the main producers of OPN during development of diet-induced obesity. OPN has been shown to amplify Ccl-2 mediated migration of macrophages [19]. Mice lacking a functional OPN gene, despite being obese, are insulin sensitive. Their adipose tissue shows decreased macrophage infiltration and reduced inflammation.

Alternatively, increased lipotoxicity may cause insulin resistance. If the amount of fuel that enters tissue cannot be dealt with, either from the oxidation, or storage, point of view, metabolites are generated which interfere with insulin action, either directly or indirectly [4], [20], [21]. Lipid metabolites, for instance ceramide species, are of particular interest here. Obesity is characterized by increased levels of FFA. Importantly, FFA have been reported to trigger pro-inflammatory responses in macrophages by acting on toll like receptors [22]–[24], and thus could act as intermediates in the vicious cycle promoting insulin resistance and inflammation. The mechanism(s) by which FFA are causing these effects are still largely unknown. FFA, such as palmitate, are essential building blocks of glycosphingolipids (GSL) [25], [26]. Several lines of evidence, obtained both *in vitro* and *in vivo*, point to a crucial role of GSL in the development of insulin resistance. Ceramide is reported to contribute to impaired insulin signalling [27]. More recently, glycosphingolipids like the ganglioside GM3 have also been shown to inhibit insulin signalling [28]–[32]. Of interest, the concentration of GM3 in cultured adipocytes is markedly increased by the pro-inflammatory cytokine TNF-α [29]. It has been demonstrated by us that inhibition of glucosylceramide synthase (GCS) activity with N-(5-adamantane-1-yl-methoxy)-pentyl-1-deoxynojirimycin (AMP-DNM) corrects insulin resistance in cultured adipocytes from obese individuals and 3T3 cells exposed to TNF-α [33].

The importance of glycosphingolipids in obesity-induced insulin resistance has recently been further illustrated by pharmacological interventions in animal models [33], [34]. We observed that inhibition of glucosylceramide synthase (GCS) activity in several rodent models of obesity reverses the insulin resistance syndrome. Feeding rodents with AMP-DNM lowered circulating glucose and HbA_1c_ levels, improved oral glucose tolerance, improved insulin sensitivity in muscle and liver and resulted in β cell preservation [33]. In addition, inhibition of GCS using (1R,2R)-nonanoic acid[2-(2,3-dihydro-benzo [1], [4] dioxin-6-yl)-2-hydroxy-1-pyrrolidin-1-ylmethyl-ethyl]-amide-L-tartaric acid salt (Genz-123346) showed comparable improved glycemic control and insulin resensitizing effects [34]. It has very recently also been reported that reduction of ceramide synthesis by inhibition of serine-palmitoyl transferase using myriocin, or via the inactivation of the dihydroceramide desaturase-1 gene, reduces insulin resistance in rodents, either evoked by glucocorticoids or saturated free fatty acids, or in obese animals [35].

Given the dramatic beneficial responses in glycemic control of obese animals following exposure to the iminosugar AMP-DNM, we investigated whether inhibiting GCS, next to a direct beneficial effect on adipocytes, also consequently was reflected by reduced inflammation in adipose tissue of leptin-deficient obese (Lep*^Ob^*) mice.

## Results

### The glucosylceramide synthesis inhibitor AMP-DNM normalizes glucose homeostasis in Lep^Ob^ mice

In the present study leptin-deficient obese Lep*^Ob^* mice were exposed to 100 mg AMP-DNM /kg bodyweight/day for 4 weeks. The inhibitor was well tolerated and caused no overt side effects. A minor reduction (p = 0.0048) in the percentage of body weight gain was noted (166±15 in the Lep*^Ob^* treated with AMP-DNM versus 202±11 in Lep*^Ob^*). Glucosylceramide, but not ceramide, was reduced in plasma from inhibitor treated animals (see table 1). Both glucose homeostasis and insulin signalling were markedly improved. Treated Lep*^Ob^* mice showed (near) normal HbA_1c_, non-fasted blood glucose concentrations and glucose clearance upon oral challenge in an oral glucose tolerance test (OGTT). Fasted insulin levels and the homeostatic model assessment (HOMA) index, which is clearly increased in Lep*^Ob^* animals, were significantly reduced upon treatment.

**Table 1:** Summary of the effects of AMP-DNM on glycosphingolipid content and blood glucose homeostasis.

	Lean	Lep*^Ob^*	Lep*^Ob^* AMP-DNM	Lean versus Lep*^Ob^*	Lep*^Ob^* versus Lep*^Ob^* AMP-DNM
Glucosylceramide (nmol/ml)	6.20±0.58	12.93±2.08	3.68±0.99	P = 0.0001	P = 0.0022
Ceramide (nmol/ml)	6.77±1.38	15.47±2.93	12.95±3.02	P = 0.0003	n.s.
HbA1C (%)	4.92±0.71	9.64±1.22	5.08±1.06	P<0.0001	P = 0.0006
Blood glucose (millimolar)	10,02±0.45	16.30±3.95	11.58±1.55	P = 0.0077	P = 0.0159
Insulin (ng/ml)	0.72±0.27	19.91±15.44	4.51±0.67	P = 0.024	P = 0.032
HOMA index	6.38±2.45	259.4±173.8	49.71±25.18	P = 0.001	P = 0.0077
OGTT (Area under the curve)	1236±114.2	2266±202.3	1580±33.58	P<0.0001	P = 0.0003

Data are means±S. E. M. Actual P values are indicated and n.s. means no significant difference (n = 5 animals per group).

### AMP-DNM improves adipocyte function in Lep^Ob^ mice

**Figure 1:**
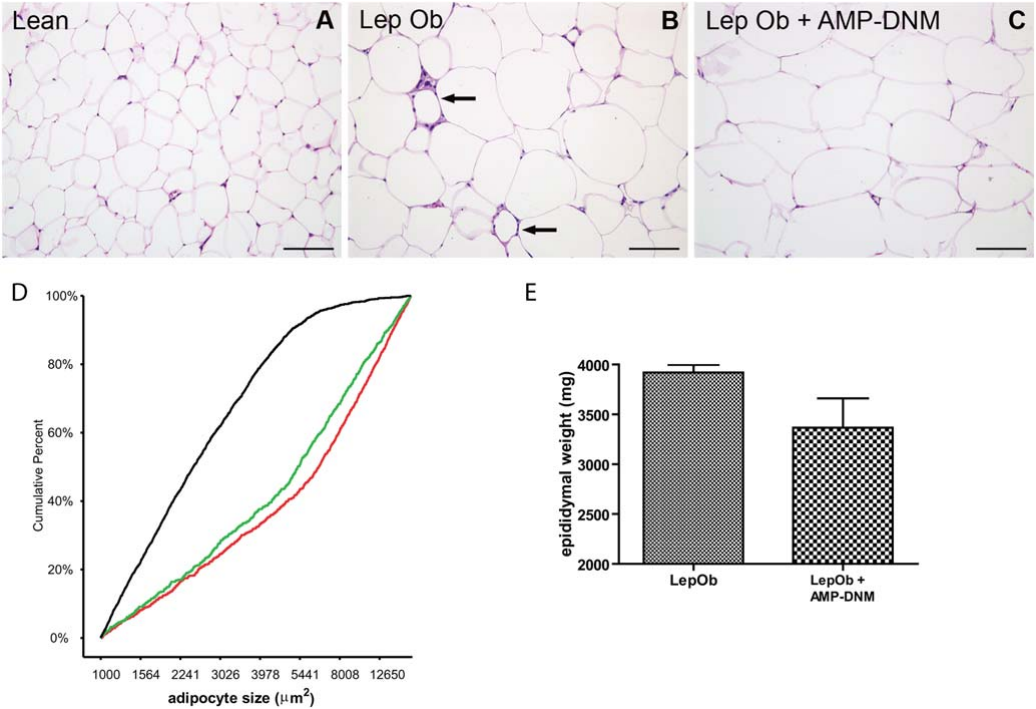
Adipose tissue analysis after lowering of glycosphingolipid content. Haematoxylin and eosin staining of adipose tissue of (A) lean mice, (B) LepOb mice and (C) LepOb mice following reduction of glycosphingolipid content. (D) Analysis of adipocyte cell size distribution in lean mice (black line), in LepOb mice (red line) and in AMP-DNM treated LepOb mice (green line). (E) EWAT weight. Data are depicted on the Y-axis as mean±S.E.M. (n = 5 per group). Actual p values are depicted in the graphs. Bars in the photographs represent 100 µm. The arrows in panel B indicate crown-like structures. For the distribution of adipocyte size at least 150 cells were analysed per animal (for details see methods).

First, the effect of AMP-DNM treatment of Lep*^Ob^* animals on adipocyte function was investigated. For this purpose, tissue slides stained with haematoxylin and eosin were examined. Adipocytes appeared larger in Lep*^Ob^* mice when compared to those in lean mice (Fig. 1B versus 1A). When analyzing the average EWAT adipocyte size, calculated per animal, we observed a significant difference (p<0.0001) between lean mice (2923±455 µm^2^) and Lep*^Ob^* mice (7992±494 µm^2^). We however did not observe a significant difference between Lep*^Ob^* animals and AMP-DNM treated animals (Fig. 1A–C). Interestingly, when studying individual adipocytes we observed a change in distribution of size (Fig. 1D). As expected the adipocytes in lean mice (black curve) were predominantly smaller than adipocytes in Lep*^Ob^* mice (red curve). Following treatment with AMP-DNM a reduction of the median adipocyte size was observed in Lep*^Ob^* mice (green curve), mainly due to a shift to the left of the 2^nd^ and 3^th^ quartiles of the distribution, reflecting reduced numbers of the large adipocytes. Adipocyte size did not normalize to sizes observed in lean mice. The distribution frequencies were analysed in detail by using a non parametric 2-tailed Kolmogorov-Smirnov Z test. The distributions are significantly different (lean versus Lep*^Ob^* p≤0.0005; Lep*^Ob^* versus Lep*^Ob^*+AMP-DNM p = 0.002). We also studied adipocytes in the omental adipose tissue (OAT) depot. In general the adipocyte sizes appeared smaller in the OAT depot when compared to the EWAT depot. We observed a comparable shift to the left with AMP-DNM and the distributions again appeared significantly different using the Kolmogorov-Smirnov Z test (lean versus Lep*^Ob^* p≤0.0005; Lep*^Ob^* versus Lep*^Ob^*+AMP-DNM p<0.0005) (data not shown). The amount of EWAT in Lep*^Ob^* animals (both pads 3920±175.1 mg) tended to be lower upon AMP-DNM treatment (both pads 336±655 mg), but the reduction failed to reach significance (p = 0.10) (Fig. 1E). No significant reduction in EWAT weight was observed when normalized for body weight.

**Figure 2:**
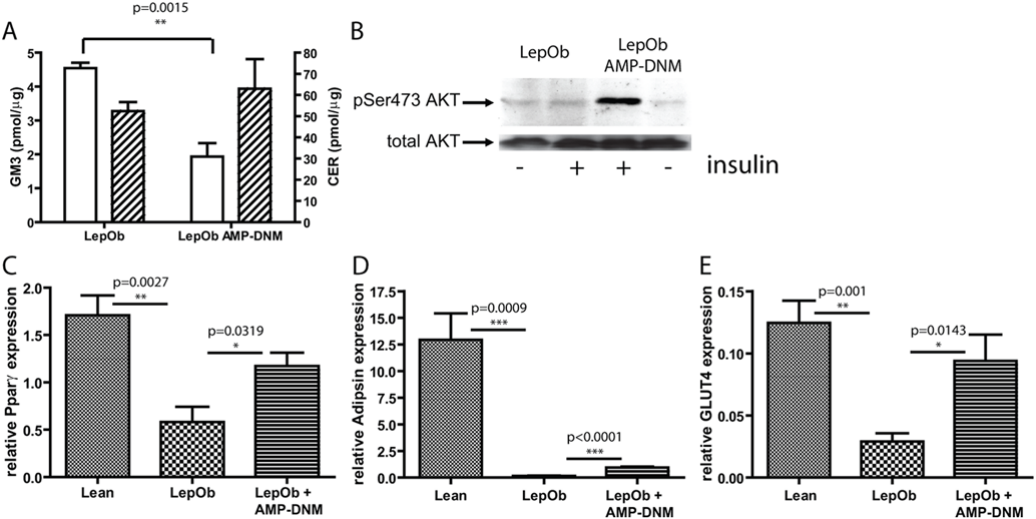
Restoration of insulin signalling and normalization of adipogenesis, following lowering of adipose tissue glycosphingolipid content (A) Reduction of GM3 concentration (open bars, left Y-axis), but not ceramide (hatched bars, right Y-axis), in adipose tissue by AMP-DNM (B) Restoration of insulin signalling by insulin as demonstrated by western blot analysis of AKT phosphorylation. Adipogenesis is improved as is demonstrated by real time PCR analysis of (C) Pparγ (D) adipsin and (E) GLUT4. In the graphs values are depicted as mean±S.E.M. (n = 5 per group), with p values indicated in the graphs.

In adipose tissue we observed a significant reduction of GM3 (4.55±0.32 pmol/µg versus 1.93±0.57 pmol/µg), again without affecting ceramide (52.42±8.61 pmol/µg versus 62.97±24.2 pmol/µg) (Fig. 2A). We next studied insulin signalling in adipocytes, which were isolated from EWAT of Lep*^Ob^* mice, or Lep*^Ob^* mice exposed to AMP-DNM. When adipocytes were stimulated ex-vivo with insulin for 10 min no phosphorylation of Akt/PKB was observed in Lep*^Ob^* derived adipocytes, whereas in the case of cells from treated animals clear phosphorylation of Akt/PKB was detected (Fig. 2B).

**Figure 3:**
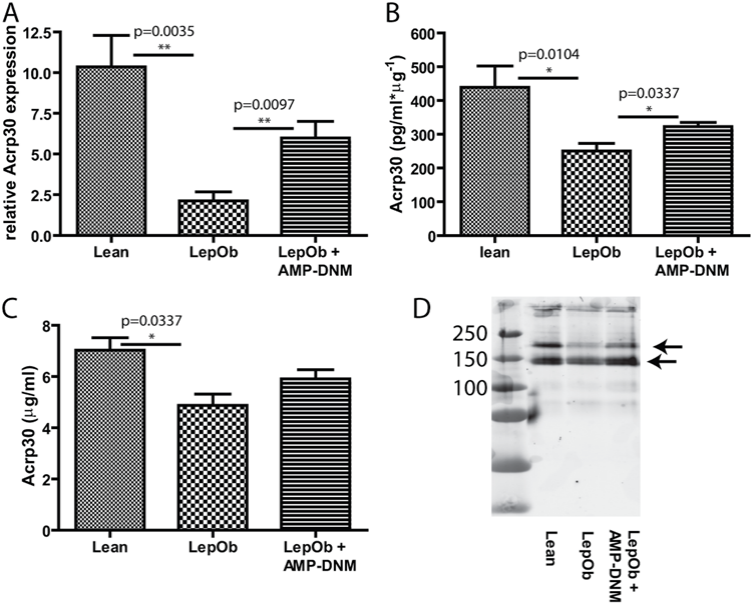
Inhibition of glycosphingolipid synthesis increases adiponectin expression. Correction of the adipokine adiponectin/Acrp30 as demonstrated by (A) real time PCR and analysis of protein levels in (B) adipose tissue and (C) plasma by ELISA. (D) Westernblot analysis of adiponectin protein in plasma. In the graphs values are depicted as mean ± S.E.M. (n = 5 per group), with p values indicated in the graphs. The arrows in panel D refer to middle molecular weight species of adiponectin.

The expression of several key genes involved in adipogenesis was analyzed. Comparing Lep*^Ob^* EWAT with comparable lean tissue, a 3-fold decrease in expression of peroxisome proliferator-activated receptor (PPAR)γ, an 80-fold decrease in expression of the adipocyte-specific serine protease adipsin and a 4-fold decrease in expression of the insulin responsive glucose transporter GLUT4 was detected using real-time PCR (Fig. 2 C–E). Decreased expression of these genes is in line with the reported loss of adipocyte differentiation and function in Lep*^Ob^* mice [36]–[42]. Interestingly, all these markers showed a significant increase when compared to untreated Lep*^Ob^* mice upon inhibition of glucosylceramide synthesis; Pparγ (2-fold), adipsin (6-fold) and GLUT-4 (3-fold), suggesting that adipogenesis is normalizing. A comparable pattern was also observed for CCAAT/enhancer binding protein (C/EBP)α and adipocyte fatty-acid-binding protein, aP2/(FABP4). On the other hand adipogenesis inhibiting Pref1 was undetectable in lean and AMP-DNM treated animals, but was detected in Lep*^Ob^* mice (data not shown). Importantly, we also observed that expression of the adipokine adiponectin/Acrp30 increased in the presence of AMP-DNM. Adiponectin RNA was 5-fold decreased in Lep*^Ob^* mice compared to normal, and increased by 3-fold in adipose tissue of AMP-DNM treated obese mice (Fig. 3A). Adiponectin protein levels in adipose tissue lysates and in serum were determined. In adipose tissue a 2-fold decrease was observed in Lep*^Ob^* mice when compared to lean mice. AMP-DNM treatment significantly increased (1.3-fold) adiponectin in adipose tissue (Fig. 3B). Serum adiponectin levels were 1.4-fold decreased in Lep*^Ob^* mice compared to lean control mice. Adiponectin showed a 1.2-fold increase in AMP-DNM treated animals, failing to reach significance (p = 0.10) (Fig. 3C). By western blot analysis we observed that several middle molecular weight species of adiponectin were lower in Lep*^Ob^* mice when compared to lean mice. Following treatment with AMP-DNM this almost normalized to levels observed in lean mice (Fig. 3D).

In conclusion, inhibition of synthesis of glucosylceramide and glycosphingolipids directly beneficially affects features of adipocytes in adipose tissue of Lep*^Ob^* mice, including insulin sensitivity and adipogenesis.

### AMP-DNM reduces macrophages in adipose tissue

**Figure 4:**
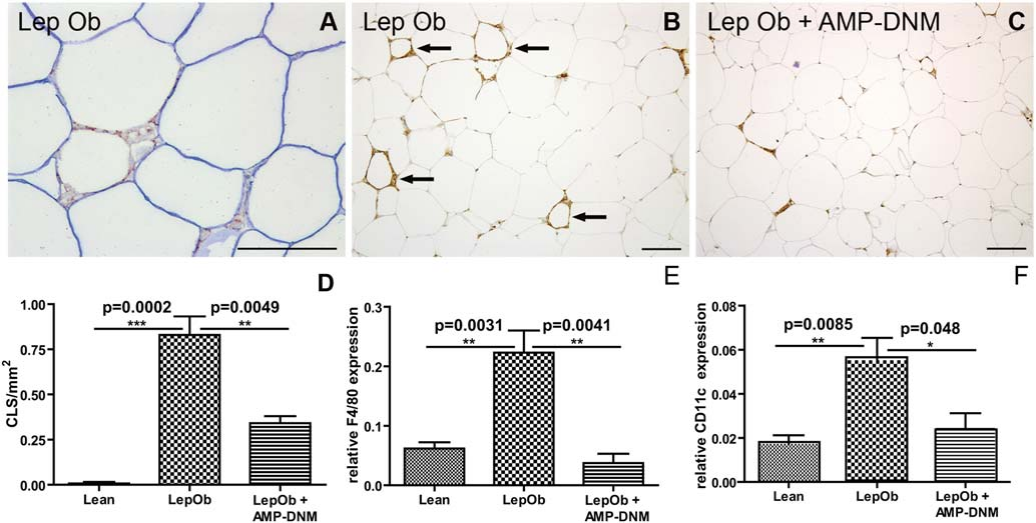
Reduced macrophage content in adipose tissue after inhibition of glycosphingolipid content with the iminosugar AMP-DNM. (A) Immunohistochemical analysis of crown-like structures in LepOb mice using double staining for F4/80 (red) and perilipin (blue). F4/80 staining in (B) LepOb and (C)AMP-DNM fed LepOb mice. (D) Quantification of crown-like structures in lean, LepOb and AMP-DNM treated LepOb mice. (E) Real time PCR analysis of F4/80 expression and (F) CD11c expression in lean, LepOb and LepOb mice fed AMP-DNM. Data are depicted on the Y-axis as mean±S.E.M. (n = 5 per group). Actual p values are depicted in the graphs. Bars in the photographs represent 100 µm.

As we clearly observed improved adipocyte function, we next investigated whether AMP-DNM treatment consequently also reduced the inflammatory status of adipose tissue in Lep*^Ob^* mice. Recently it has been demonstrated that non-viable adipocytes in leptin receptor-deficient mice and obese human subjects lack perilipin protein [15], [43]. Using immunohistochemistry double staining, we confirmed this also in Lep*^Ob^* mice. The crown-like structures showed F4/80 positive adipose tissue macrophages (ATM) surrounding an adipocyte, which lost perilipin positive signal (Fig. 4A). As expected, we observed F4/80^+^ crown-like structures, i.e. ATM surrounding dead adipocytes, in tissue specimens of obese animals (Fig. 1B, Fig. 4B indicated with arrows). In tissue specimens of lean animals these structures could not be detected and they were hardly detectable in Lep*^Ob^* mice treated with the iminosugar AMP-DNM (Fig. 1A, C and Fig. 4C). Next, we analyzed the number of F4/80 positive crown-like structures in Lep*^Ob^* animals and in AMP-DNM fed Lep*^Ob^* animals. Representative images are depicted in Fig. 4B and C (indicated by arrows is the F4/80 staining, Fig. 4B). A graph summarizing the quantitative analysis of crown-like structures is depicted in Fig. 4D. Real time PCR showed a consistent reduction in F4/80 mRNA in the AMP-DNM fed Lep*^Ob^* mice. F4/80 mRNA was approximately 4-fold increased in Lep*^Ob^* adipose tissue and normalized in treated Lep*^Ob^* mice (Fig. 4E). Furthermore, CD11c gene expression, which is found on recruited proinflammatory ATM, normalized following treatment with AMP-DNM (Fig. 4F) [24], [44].

These findings indicate that AMP-DNM treatment results in less crown-like structures and near normalisation of ATM numbers in EWAT of Lep*^Ob^* mice.

### Anti-inflammatory effect of AMP-DNM

**Figure 5:**
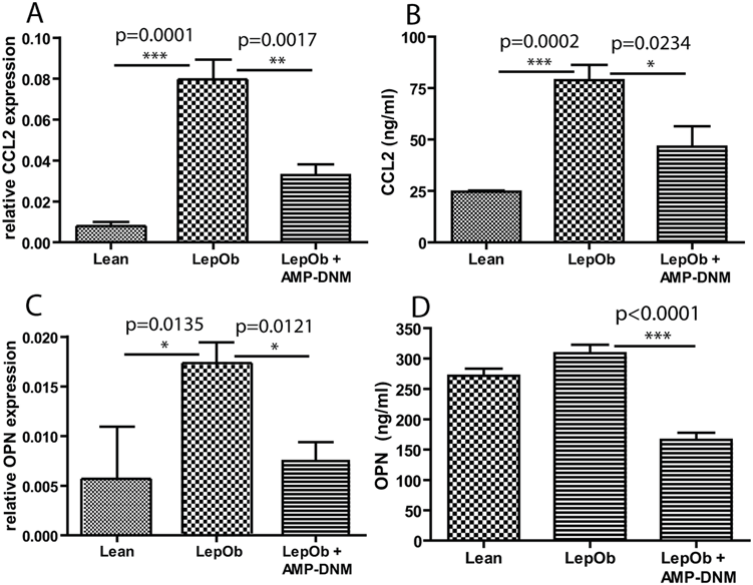
The chemo attractants Ccl2 and OPN are reduced in adipose tissue and in the circulation following treatment with AMP-DNM. (A) Real time PCR analysis of Ccl2 expression in adipose tissue (B) Analysis of Ccl2 protein by ELISA in plasma (C) Real time PCR analysis of OPN expression in adipose tissue (D) Analysis of OPN protein by ELISA in plasma. Data are depicted on the Y-axis as mean±S.E.M. (n = 5 per group). Actual p values are depicted in the graphs.

Next, we studied inflammation in adipose tissue in more detail by analysing gene expression profiles in EWAT from lean C57BL/6J control mice, Lep*^Ob^* mice and Lep*^Ob^* mice treated with AMP-DNM. Q-PCR arrays were used, focussing on inflammatory cytokines and receptors (analyzed genes are listed in Supplementary data table S1). The most regulated genes are shown in table 2. AMP-DNM treatment of Lep*^Ob^* mice most pronouncedly effected expression of osteopontin/OPN (on average 20-fold increased in Lep*^Ob^* and 2.5-fold reduced upon treatment) and Ccl2 (on average 9-fold increased in Lep*^Ob^* and 2.5- fold reduced upon treatment (Fig. 5A, C). As measured by ELISA, Ccl2 in serum of Lep*^Ob^* mice was also found to be lower upon treatment (Fig. 5B). OPN in serum of Lep*^Ob^* mice was hardly increased, but reduced by AMP-DNM treatment (Fig. 5D). The mRNA encoding the chemokine Cxcl11 was on average 10-fold reduced in adipose tissue of Lep*^Ob^* mice. In treated animals its concentration was 3-fold increased (see table 2). Table 3 shows the genes of which expression did increase in Lep*^Ob^* mice, but was not affected by AMP-DNM. Ccl-8 is the most striking in this respect (on average 12-fold increased in obese animals but not changed by inhibitor treatment). Furthermore, we observed that expression of TNFα increased in Lep*^Ob^* adipose tissue, but AMP-DNM treatment did not correct this. Table 4 shows the genes of which expression was not changed in Lep*^Ob^*, but is affected by AMP-DNM treatment. For none of the genes upregulated by AMP-DNM, a clear anti-inflammatory action is documented.

**Table 2:** Inflammatory mediators induced in Lep*^Ob^* mice and reverted by AMP-DNM.

Lep*^Ob^* versus lean control			Lep*^Ob^* AMP-DNM versus Lep*^Ob^*		
Symbol	P value	Fold up/down	Symbol	P value	Fold down/up
Spp1/OPN	0,014	20,2	Spp1	0,012	−2,5
Ccl2	0,000008	9,1	Ccl2	0,0017	−2,4
Il10	0,00017	6,9	Il10	0,0054	−1,9
Il3	0,0118	5,8	Il3	*0,074*	−3,6
Ccl7	0,000024	5,2	Ccl7	0,046	−1,8
Ccl12	0,000043	4,2	Ccl12	*0,062*	−1,6
Ccl9	0,000083	3,0	Ccl9	0,0069	−1,9
Ccr5	0,0010	2,4	Ccr5	0,0047	−1,8
Itgam	0,0022	1,8	Itgam	0,039	−1,5
Tnfrsf1b	0,0031	1,6	Tnfrsf1b	0,022	−1,3
Ccr4	0,010	−3,0	Ccr4	0,030	1,7
Ccl24	0,000004	−3,7	Ccl24	*0,056*	1,6
Cxcl11	0,000043	−9,7	Cxcl11	0,013	3,1

Data depicted are average up -or down regulated inflammatory genes when Lep*^Ob^* mice are compared to lean control mice (left three columns), which are reverted by treatment with AMP-DNM (right three columns). Actual P values are indicated, in italics P values showing a trend, not reaching significance.

**Table 3:** Inflammatory mediators induced in Lep*^Ob^* mice but not reverted by AMP-DNM.

Lep*^Ob^* versus lean control			Lep*^Ob^* AMP-DNM versus Lep*^Ob^*		
Symbol	P value	Fold up/down	Symbol	P value	Fold down/up
Ccl8	0,000011	11,6	Ccl8	0,33	−1,4
Il1r2	0,0066	4,1	Il1r2	0,16	−1,5
Il13	0,00020	3,6	Il13	0,14	−1,4
Ccl3	0,0026	2,9	Ccl3	0,95	−1,0
Ccl4	0,0018	2,5	Ccl4	0,12	−1,3
Cxcl10	0,0018	2,5	Cxcl10	0,86	1,1
Gusb	0,000001	2,4	Gusb	0,088	−1,3
Itgb2	0,0010	2,4	Itgb2	0,95	1,0
Cxcl12	0,0051	2,0	Cxcl12	0,12	−1,3
Ccl1	*0,052*	2,0	Ccl1	0,53	1,3
Tnf	0,0049	1,9	Tnf	0,37	1,1
Casp1	0,030	1,6	Casp1	0,85	−1,0
Il6st	0,0032	−1,6	Il6st	0,91	−1,0
Ccl25	0,049	−1,7	Ccl25	0,37	−1,3
Hspcb	0,016	−1,7	Hspcb	0,74	−1,1
C3	0,00019	−1,8	C3	0,85	1,0
Il16	0,0054	−1,8	Il16	0,091	1,4
Il18	0,0017	−2,0	Il18	0,79	1,1
Abcf1	0,000006	−2,0	Abcf1	0,28	−1,1
Gpr2	0,015	−2,1	Gpr2	0,86	1,1
Ccr8	0,039	−2,2	Ccr8	0,40	1,3

Data depicted are average up -or down regulated inflammatory genes when Lep*^Ob^* mice are compared to lean control mice (left three columns), which are not changed by treatment with AMP-DNM (right three columns). Actual P values are indicated.

**Table 4:** Inflammatory mediators not induced in Lep*^Ob^* but induced by AMP-DNM.

Lep*^Ob^* versus lean control			Lep*^Ob^* AMP-DNM versus Lep*^Ob^*		
Symbol	P value	Fold up/down	Symbol	P value	Fold down/up
Ccr7	0,31	−1,9	Ccr7	0,0028	2,8
Ccl11	0,46	−1,1	Ccl11	0,017	1,8
Ccl22	0,65	−1,2	Ccl22	0,0076	1,7
Cxcr3	0,91	1,0	Cxcr3	0,046	1,6

Data depicted are average values of unchanged inflammatory genes when Lep*^Ob^* mice are compared to lean control mice (left three columns), which are induced by treatment with AMP-DNM (right three columns). Actual P values are indicated.

In conclusion, two important proteins involved in the recruitment of ATM to adipose tissue and local inflammation, Ccl2 and OPN, were down-regulated in adipose tissue after treatment with AMP-DNM.

## Discussion

The present study reveals for the first time that partial inhibition of glucosylceramide biosynthesis, and subsequent glycosphingolipids, not only restores insulin sensitivity of adipocytes in Lep*^Ob^* mice, but also improves adipocyte function and consequently reduces the number of macrophages (crown-like structures) and local inflammation.

EWAT weight showed a trend towards reduction in AMP-DNM fed Lep*^Ob^* mice. Possibly, prolonged exposure to AMP-DNM could result in a more significant reduction. Importantly, the unresponsiveness of the insulin receptor towards insulin was found to be reversed in adipocytes isolated from adipose tissue of AMP-DNM treated Lep*^Ob^* mice. Other relevant changes in the adipose tissue were also improved by AMP-DNM treatment. Several genes whose expression is reduced in adipose tissue of obese Lep*^Ob^* mice, such as PPARγ, adipsin, GLUT4, C/EBPα and aP2/FABP4 showed an increase following AMP-DNM treatment. Pref-1, which negatively regulates adipogenesis, was not detected in lean, or AMP-DNM treated Lep^Ob^ mice. Detailed analysis of adipocyte size revealed that AMP-DNM treatment reduced adipocyte size, both in EWAT and OAT. These findings suggest at least partial restoration of normal adipogenesis. Interestingly, we also found an increase in expression of the adipokine adiponectin at the level of RNA and protein in adipose tissue. In plasma adiponectin levels tended to increase as well and western blot analysis revealed an increase of intermediate molecular weight forms of adiponectin in the presence of AMP-DNM. Adiponectin has been shown to improve whole body insulin sensitivity and shows anti-inflammatory properties. *In vitro*, monocyte adherence to endothelial cells is reduced by adiponectin as endothelial cell adhesion molecules such as intercellular adhesion molecule-1 and vascular cell adhesion molecule-1 are suppressed. In addition, pro-inflammatory mediators such as Ccl2 are suppressed in macrophages by adiponectin [36], [37], [45], [46]. Recently, it was reported that modest over expression of circulating adiponectin in Lep*^Ob^* mice completely rescued the diabetic phenotype in Lep*^Ob^* mice. In addition, increased expression of PPARγ target genes and reduced ATM infiltration in adipose tissue and systemic inflammation was observed. These mice however were morbidly obese, with more adipose tissue than their Lep*^Ob^* littermates [5]. The effects induced by over expression of adiponectin resemble those invoked by AMP-DNM. The noted corrections in adiponectin in Lep*^Ob^* after AMP-DNM treatment may therefore significantly contribute to the remarkable improvement in total body glucose homeostasis in AMP-DNM treated mice. The effect of AMP-DNM points towards improved expandability of adipose tissue of the mice.

In line with the restored adipose tissue function we observed that AMP-DNM treatment reduces inflammation in EWAT as well. Adipose tissue of obese individuals is rich in perilipin-negative, predominantly dead, adipocytes surrounded by macrophages, giving rise to so-called crown-like structures [15], [43]. AMP-DNM treatment of Lep*^Ob^* mice was found to result in less perilipin-negative adipocytes in EWAT, and concomitantly less surrounding ATM. We observed reduced CLS formation following treatment with APM-DNM and in line with this finding macrophage-specific F4/80 RNA expression was reduced in treated Lep*^Ob^* mice to levels observed in lean controls. Importantly, also CD11c expression normalized and this marker is expressed by the inflammatory macrophages, which are recruited to adipose tissue and contribute to insulin resistance [24], [44], [47], [48]. Evidence presented here suggests improved adipogenesis. This in turn can also contribute to reduced inflammation taking into account that preadipocytes produce more inflammatory mediators compared to mature adipocytes. More detailed analysis of EWAT revealed that several genes whose expression is increased in Lep*^Ob^* mice compared to lean control mice, decreased following lowering of glycosphingolipid content. The most prominently corrected genes were Ccl2 and OPN. Importantly, both these proteins are considered to be essential mediators in the recruitment of inflammatory macrophages towards adipose tissue and to be involved in the induction of insulin resistance [16], [19]. OPN is specifically induced in ATM during high fat diet induced obesity. Ablation of OPN does not interfere with obesity itself, but reduces ATM content, inflammation and improves insulin sensitivity of adipose tissue. In addition, it has been demonstrated that OPN promotes Ccl2-mediated migration of macrophages to adipose tissue [19]. Increased levels of OPN have also been reported in obese human subjects. Interestingly, following weight loss after a dietary intervention a significant down-regulation op OPN protein was observed [49]. The reduced number of F4/80 positive macrophages by AMP-DNM fits well with the finding that macrophage-derived chemo-attractants Ccl2 and OPN are concomitantly reduced. Interestingly, an anti-inflammatory effect of AMP-DNM has previously been found in a hapten-induced model of colitis, which possibly acts through macrophages. Amongst others reduced cellular infiltration and myeloperoxidase activity were found in colon and within lesions lower IFNγ and IL-18 production was detected [50].

At a dose of 25 mg/kg/day AMP-DNM was found to have no significant effect on food intake in Lep*^Ob^* mice [33]. In the present study a minor reduction in food intake was noted in the animals treated with 100 mg/kg/day AMP-DNM. We therefore can not rule out that on top of the beneficial effect of more marked glycosphingolipid lowering, other factors add to this. It might be that at the well tolerated higher dose of 100 mg/kg/day, AMP-DNM also corrects appetite, energy expenditure, or brown adipose tissue. Follow-up investigations are needed to test these possibilities.

**Figure 6:**
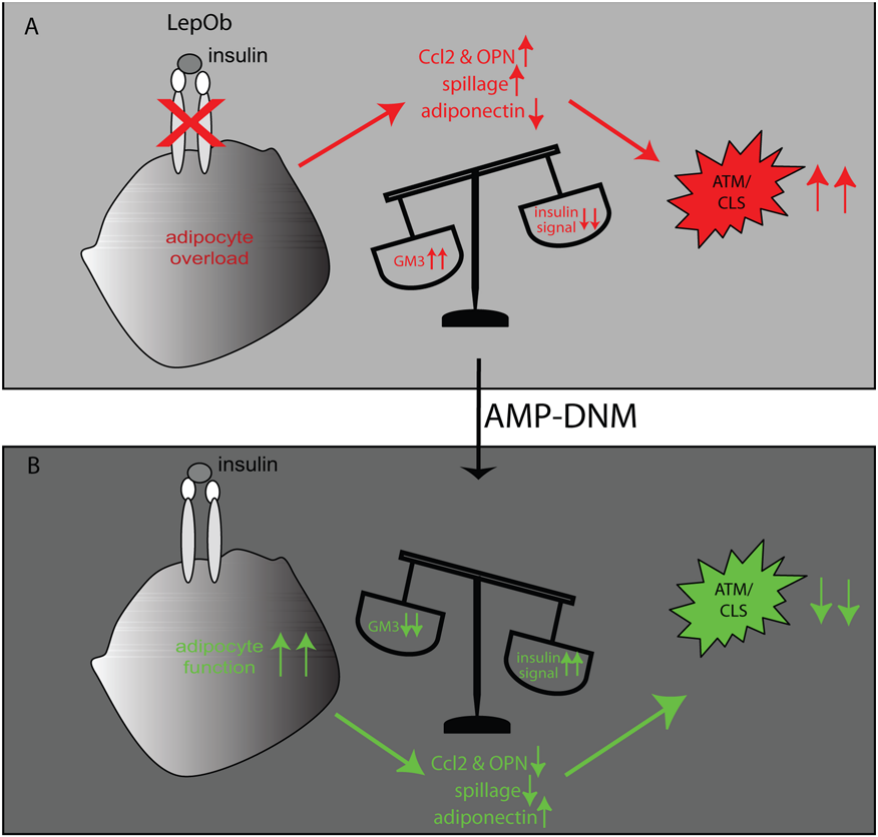
Proposed mechanism of action of AMP-DNM. (A) Pathological GM3 levels inhibit insulin receptor signalling. Adipocyte dysfunction and ultimately its death results in production of inflammatory mediators, reduction of adiponectin production and spillage of lipotoxic content, which triggers macrophage activation and crown-like structure (CLS) formation. (B). Lowering of glycosphingolipids, especially GM3, restores insulin signalling, improves adipogenesis, increases adiponectin and reduces lipotoxic spillage, all leading to less inflammation and disappearance of CLS.

Fig. 6 represents a model giving a possible explanation for our observations regarding the effects of glycosphingolipid lowering by AMP-DNM in adipose tissue of obese Lep*^Ob^* mice. AMP-DNM inhibits synthesis of glucosylceramide and subsequently GM3 formation, which results in restoration of insulin signalling. In addition, adipogenesis and adiponectin production are improved, all resulting in improved adipocyte function. As a consequence inflammation is reduced because the trigger, a dysfunctional adipocyte undergoing cell death with concomitant macrophage activation, is lost. This fits our finding of reduced numbers of crown-like structures and the reduction of the macrophage chemoattractants Ccl2 and OPN. The noted improvement in adipose tissue imposed by AMP-DNM treatment might add to the overall corrections in tissues such as muscle and liver [33].

In conclusion, we demonstrate in Lep*^Ob^* mice that AMP-DNM exerts multiple beneficial effects on adipose tissue. Reduction of glycosphingolipid content of adipocytes promotes their insulin sensitivity directly and stimulates adipogenesis. As a consequence ATM content of adipose tissue is reduced and this contributes to a less deleterious environment.

## Materials and Methods

### Animals

C57BL/6J control mice and leptin-deficient obese (Lep*^Ob^*) mice (C57BL/6J background) were obtained from Harlan (Horst, the Netherlands). Animals, n = 5 per group unless stated differently, were fed a commercial chow diet (AM-II) with or without 100 mg AMP-DNM/kg bodyweight per day for four weeks (Arie Blok BV, Woerden, the Netherlands). AMP-DNM has been synthesized as described previously [33], [51]. Studies were initiated using 7 week old male mice. Approval for the study was obtained from the local ethical committee for animal experiments.

### Plasma and tissue sampling

Blood samples were collected by either tail vein or retro orbital plexus puncture. After four weeks animals were sacrificed and a large blood sample was collected by cardiac puncture. Subsequently, tissues were quickly removed and either used directly, or immediately placed in liquid nitrogen or fixed in formalin for further analysis.

### Measurement of blood glucose, HbA_1c_, insulin and oral glucose tolerance

Blood glucose levels were determined in plasma of non-fasted animals using a Glucometer (Ascensia Elite, Bayer A.G., Leverkusen, Germany). HbA_1c_ levels were measured in whole blood of non- fasted animals using a single measurement A1C now device (Metrika, Sunnyvale, USA). Fasted insulin levels were determined by ELISA (Crystal Chem Inc, USA). Oral Glucose Tolerance Tests (OGTT) were performed in fasted animals (4 h) by gavage of glucose (500 mg glucose/kg body weight). Blood glucose values were measured immediately before and 20, 40, 60, 90 and 120 min after glucose loading. Area under the curve (AUC) (arbitrary units per minute) was determined for individual animals.

### Analysis of lipids

Lipids were extracted according to Folch et al. [52]. Ceramide and glucosylceramide were determined by high-performance liquid chromatography (HPLC) analysis of orthophtaldehyde-conjugated lipids according to a procedure previously described [53]. Deacylation of lipids was performed in 0.5 ml 0.1 mol/l NaOH in methanol in a microwave oven (CEM microwave Solids/Moisture System SAM-155). Deacylated glycolipids were derivatized on line for 30 min with O-phtaldehyde. Analysis was performed using an HPLC system (Waters Associates, Milford, USA) and a Hypersil BDS C_18_ 3 µ, 150×4.6-mm reverse-phase column (Alltech Inc., USA).

GM3 was detected by analysis of the acidic glycolipid fraction obtained by Folch extraction as has been described previously with slight modifications [54]. Gangliosides were desalted on a disposable SPE C18 column (Bakerbond, Mallinckrodt Baker Inc., Phillipsburg, NJ, USA) as described by Kundu [55] and quantified following release of oligosaccharides from glycosphingolipids by ceramide glycanase (Recombinant endoglycoceramidase II, Takara Bio Inc., Otsu, Shiga, Japan) digestion. The enzyme was used according to the manufacturer's instructions. Released oligosaccharides were labeled at their reducing end with the fluorescent compound anthranilic acid (2-aminobenzoic acid), prior to analysis using normal-phase high-performance liquid chromatography [56]. Throughout the procedure trisialoganglioside-GT1b (Sigma, St Louis, Mo, USA) was used as an internal standard.

### Ex vivo analysis of insulin signalling in freshly isolated adipocytes

Epididymal white adipose tissue (EWAT) of Lep*^Ob^* mice and AMP-DNM fed Lep*^Ob^* mice was surgically removed and exposed to collagenase treatment. Collagenase VIII (Sigma-Aldrich Chemie BV, Zwijndrecht, The Netherlands) was diluted at 1 mg/ml in buffer (1×HBSS, 20 mM Hepes (pH = 7.4), 4.17 mM Sodiumbicarbonate and 2% fatty acid free BSA). Fat tissue was cut into small pieces and incubated for 15 min at 37°C. Next, adipocytes were enriched for by density centrifugation for 5 min at 250×*g*. Floating adipocytes were washed for three times. Subsequently, adipocyte-enriched cell suspensions were stimulated with or without insulin (100 nM, Sigma-Aldrich) for 10 min followed by cell lysis in RIPA (150 mM NaCl, 10 mM Tris pH 7.2, 0.1% SDS, 1% Triton, 1% deoxycholate, 5 mM EDTA) buffer, supplemented with protease inhibitors (Roche, 1 tablet per 20 ml of lysis buffer). Equal amounts of total protein in lysates were separated by SDS-PAGE, followed by standard ECL on immunoblots using anti-pSer473 Akt, and anti-total Akt (Cell Signaling Technology, Inc., USA) and goat anti-mouse peroxidase (Biorad) [33].

### Immunohistochemistry of adipose tissue macrophages and adipocytes

EWAT was fixed in buffered formalin and embedded in paraffin. Deparaffinized sections (4 µm) were stained with haematoxylin-eosin. After quenching of endogenous peroxidase activity by 0.3% H_2_O_2_ in methanol and blocking of free protein-binding sites with 5% normal goat serum, sections were immunostained for macrophages using rat IgG2b anti-mouse F4/80 monoclonal antibody (AbD Serotec, Oxford, UK). In some cases, doublestaining was performed for perilipin using a monospecific guinea pig polyclonal antibody (Progen, Heidelberg, Germany). Specific secondary antibodies were peroxidase (HRP)-conjugated goat anti-rat IgG (SouthernBiotech, Birmingham, AL) and biotinylated goat anti-guinea pig IgG (Chemicon, Temecula, CA), respectively. The latter was followed by alkaline phosphatase-conjugated streptavidin (DAKO, Glostrup, Denmark). Bound HRP activity was visualized using either diaminobenzidine or NovaRed (Vector Laboratories, Burlingame, CA) as substrates; Vector Blue was used to detect AP activity. Hematoxylin-and-eosin-stained 6-µm-thick sections of EWAT and of omental adipose tissue (OAT) were analyzed using a Leica DM5000B microscope with 10× objective (Leica, Rijswijk, The Netherlands) and Image Pro Plus 5.02 software (Media Cybernetics, Bethesda, MD) to measure the cross-sectional surface area of individual adipocytes as an indicator of adipocyte cell size. To eliminate analysis of vessels and interstitial cells a lower threshold of 1000 µm^2^ was applied. Of each mouse at least 150 adipocytes were measured in OAT and in EWAT. Differences between average values of groups of mice were statistically evaluated with the Kruskal Wallis rank sum test and the Mann-Whitney U test. In addition, data are presented as cumulative frequency distributions. Differences were evaluated applying the 2-tailed Kolmogorov-Smirnov Z test. Statistical analysis was performed using SPSS 16.0 (SPSS Inc., Chicago, Il).

Crownlike structures (CLS) were identified in EWAT sections as single adipocytes surrounded by at least 4 F4/80-positive macrophages; CLS were counted in at least 25 mm^2^ of EWAT surface area.

### RNA extraction and real time PCR

Total RNA was extracted from EWAT using TRIZOL (Invitrogen, Breda, The Netherlands) and the nucleospin II extraction kit (Macherey-Nagel GmbH, Duren, Germany). RNA concentrations were measured using the Nanodrop Spectrophotometer (Nanodrop Technologies, USA). Equal amounts of RNA were used to synthesize cDNA, according to the manufacturer's method (Invitrogen). cDNA was diluted 10× prior to gene-specific analysis by real-time RT-PCR using an iCycler MyiQTM system (Biorad Laboratories, Hercules, USA). Expression levels were normalized to ribosomal phosphoprotein 36B4. As primers were used: 36B4 forward primer ggacccgagaagacctcctt, reverse primer gcacatcactcagaatttcaatgg; Pparγ forward primer ggaagaccactcgcattcctt, reverse primer tcgcactttggtattcttggag; GLUT4 forward primer ctcatgggcctagccaatg, reverse primer gggcgatttctcccacatac; Adipsin forward primer tccgcccctgaaccctacaa, reverse primer taatggtgactaccccgtca; ACRP30/adiponectin forward primer gctcctgctttggtccctccac, reverse primer gcccttcagctcctgtcattcc; F4/80 forward primer ctttggctatgggcttccagtc, reverse primer gcaaggaggacagagtttatcgtg. CD11c/Itgax forward primer ctggatagcctttcttctgctg, reverse primer gcacactgtgtccgaactc. C/EBPα forward primer ttacaacaggccaggtttcc, reverse primer ctctgggatggatcgattgt. aP2/FABP4 forward primer gcgtggaattcgatgaaatca, reverse primer cccgccatctagggttatga. Pref1 forward primer agctggcggtcaatatcatc, reverse primer agctctaaggaaccccggta. Data were analysed using the delta C_t_ method [57]. Alternatively, RT^2^ Profiler™ PCR arrays were used to monitor 84 mouse inflammatory cytokine and receptor genes with build in house hold genes (Super Array Bioscience Corporation, MD, USA). For cDNA synthesis the RT^2^ PCR array first strand kit was used according to the instruction of the manufacturer (Super Array Bioscience Corporation). In supplementary data table S1, the analyzed genes are listed.

### ELISA

Ccl2/Mcp-1, osteopontin/OPN, and adiponectin/Acrp30 were measured by ELISA according to the instructions of the manufacturer (R&D systems, Inc., Minneapolis, USA). Adiponectin was analysed in plasma samples of lean, Lep*^Ob^* and AMP-DNM treated Lep*^Ob^* mice by westernblot, using goat anti-mouse adiponectin antibody (R&D systems).

### Statistical analysis

Values presented in figures represent means±S.E.M. Statistical analysis of two groups was assessed by Student's t-test (two tailed). Level of significance is depicted in the figures with the actual P values, p values<0.05 were considered significant.

## Supporting Information

Table S1 Genes analyzed in relation to inflammation
(0.13 MB DOC)
