# Supplementary material for: Reducing Glycosphingolipid Content in Adipose Tissue of Obese Mice Restores Insulin Sensitivity, Adipogenesis and Reduces Inflammation
Source: PLoS One. 2009 Mar 23;4(3):e4723. doi: 10.1371/journal.pone.0004723 (PMC2654925; doi:10.1371/journal.pone.0004723)
Supplement: Table S1 — Genes analyzed in relation to inflammation (0.13 MB DOC) [file pone.0004723.s001.doc]

| **RefSeq** | **Symbol** | **Description** | **Gene Name** |
| --- | --- | --- | --- |
| NM_013854 | Abcf1 | ATP-binding cassette, sub-family F (GCN20), member 1 | AU041969/Abc50 |
| NM_009744 | Bcl6 | B-cell leukemia/lymphoma 6 | Bcl5 |
| NM_007551 | Blr1 | Burkitt lymphoma receptor 1 | CXC-R5/CXCR-5 |
| NM_009778 | C3 | Complement component 3 | AI255234/ASP |
| NM_009807 | Casp1 | Caspase 1 | ICE/Il1bc |
| NM_011329 | Ccl1 | Chemokine (C-C motif) ligand 1 | BF534335/I-309 |
| NM_011330 | Ccl11 | Small chemokine (C-C motif) ligand 11 | Scya11/eotaxin |
| NM_011331 | Ccl12 | Chemokine (C-C motif) ligand 12 | MCP-5/Scya12 |
| NM_011332 | Ccl17 | Chemokine (C-C motif) ligand 17 | ABCD-2/Scya17 |
| NM_011888 | Ccl19 | Chemokine (C-C motif) ligand 19 | CKb11/ELC |
| NM_011333 | Ccl2 | Chemokine (C-C motif) ligand 2 | AI323594/HC11 |
| NM_016960 | Ccl20 | Chemokine (C-C motif) ligand 20 | CKb4/LARC |
| NM_009137 | Ccl22 | Chemokine (C-C motif) ligand 22 | ABCD-1/DCBCK |
| NM_019577 | Ccl24 | Chemokine (C-C motif) ligand 24 | CKb-6/MPIF-2 |
| NM_009138 | Ccl25 | Chemokine (C-C motif) ligand 25 | AI852536/CKb15 |
| NM_011337 | Ccl3 | Chemokine (C-C motif) ligand 3 | AI323804/G0S19-1 |
| NM_013652 | Ccl4 | Chemokine (C-C motif) ligand 4 | Act-2/MIP-1B |
| NM_013653 | Ccl5 | Chemokine (C-C motif) ligand 5 | MuRantes/RANTES |
| NM_009139 | Ccl6 | Chemokine (C-C motif) ligand 6 | MRP-1/Scya6 |
| NM_013654 | Ccl7 | Chemokine (C-C motif) ligand 7 | MCP-3/Scya7 |
| NM_021443 | Ccl8 | Chemokine (C-C motif) ligand 8 | 1810063B20Rik/AB023418 |
| NM_011338 | Ccl9 | Chemokine (C-C motif) ligand 9 | CCF18/MRP-2 |
| NM_009912 | Ccr1 | Chemokine (C-C motif) receptor 1 | Cmkbr1/Mip-1a-R |
| NM_009915 | Ccr2 | Chemokine (C-C motif) receptor 2 | CC-CKR-2/CCR2A |
| NM_009914 | Ccr3 | Chemokine (C-C motif) receptor 3 | CC-CKR3/CKR3 |
| NM_009916 | Ccr4 | Chemokine (C-C motif) receptor 4 | Cmkbr4/LESTR |
| NM_009917 | Ccr5 | Chemokine (C-C motif) receptor 5 | AM4-7/CD195 |
| NM_009835 | Ccr6 | Chemokine (C-C motif) receptor 6 | Cmkbr6 |
| NM_007719 | Ccr7 | Chemokine (C-C motif) receptor 7 | CD197/Cdw197 |
| NM_007720 | Ccr8 | Chemokine (C-C motif) receptor 8 | Cmkbr8/mCCR8 |
| NM_009913 | Ccr9 | Chemokine (C-C motif) receptor 9 | Cmkbr10/GPR-9-6 |
| NM_007768 | Crp | C-reactive protein, petaxin related | AI255847 |
| NM_009142 | Cx3cl1 | Chemokine (C-X3-C motif) ligand 1 | AB030188/ABCD-3 |
| NM_008176 | Cxcl1 | Chemokine (C-X-C motif) ligand 1 | Fsp/Gro1 |
| NM_021274 | Cxcl10 | Chemokine (C-X-C motif) ligand 10 | C7/CRG-2 |
| NM_019494 | Cxcl11 | Chemokine (C-X-C motif) ligand 11 | CXC11/H174 |
| NM_021704 | Cxcl12 | Chemokine (C-X-C motif) ligand 12 | AI174028/PBSF |
| NM_018866 | Cxcl13 | Chemokine (C-X-C motif) ligand 13 | ANGIE2/Angie |
| NM_011339 | Cxcl15 | Chemokine (C-X-C motif) ligand 15 | Scyb15/lungkine |
| NM_019932 | Cxcl4 | Chemokine (C-X-C motif) ligand 4 | Pf4/Scyb4 |
| NM_009141 | Cxcl5 | Chemokine (C-X-C motif) ligand 5 | AMCF-II/ENA-78 |
| NM_008599 | Cxcl9 | Chemokine (C-X-C motif) ligand 9 | BB139920/CMK |
| NM_009910 | Cxcr3 | Chemokine (C-X-C motif) receptor 3 | Cd183/Cmkar3 |
| XM_894898 | Gpr2 | 7-transmembrane G-protein coupled receptor 2 (Gpr2) | Cmkbr9/Ccr10 |
| NM_008337 | Ifng | Interferon gamma | IFN-g/IFN-gamma |
| NM_010548 | Il10 | Interleukin 10 | CSIF/Il-10 |
| NM_008348 | Il10ra | Interleukin 10 receptor, alpha | AW553859/CDw210 |
| NM_008349 | Il10rb | Interleukin 10 receptor, beta | 6620401D04Rik/AI528744 |
| NM_008350 | Il11 | Interleukin 11 | IL-11 |
| NM_008355 | Il13 | Interleukin 13 | Il-13 |
| NM_133990 | Il13ra1 | Interleukin 13 receptor, alpha 1 | AI882074/CD213a1 |
| NM_008357 | Il15 | Interleukin 15 | AI503618 |
| NM_010551 | Il16 | Interleukin 16 | mKIAA4048 |
| NM_019508 | Il17b | Interleukin 17B | 1110006O16Rik/1700006N07Rik |
| NM_008360 | Il18 | Interleukin 18 | Igif/Il-18 |
| NM_010554 | Il1a | Interleukin 1 alpha | Il-1a |
| NM_008361 | Il1b | Interleukin 1 beta | IL-1beta/Il-1b |
| NM_019450 | Il1f6 | Interleukin 1 family, member 6 | Fil1/IL-1H1 |
| XM_130058 | Il1f8 | Interleukin 1 family, member 8 | 2310043N20Rik |
| NM_008362 | Il1r1 | Interleukin 1 receptor, type I | CD121a/CD121b |
| NM_010555 | Il1r2 | Interleukin 1 receptor, type II | CD121b/Il1r-2 |
| NM_021380 | Il20 | Interleukin 20 | Zcyto10 |
| NM_008368 | Il2rb | Interleukin 2 receptor, beta chain | CD122/IL-15Rbeta |
| NM_013563 | Il2rg | Interleukin 2 receptor, gamma chain | CD132/[g]c |
| NM_010556 | Il3 | Interleukin 3 | Csfmu/Il-3 |
| NM_021283 | Il4 | Interleukin 4 | IgG1/Il-4 |
| NM_008370 | Il5ra | Interleukin 5 receptor, alpha | CD125/CDw125 |
| NM_010559 | Il6ra | Interleukin 6 receptor, alpha | CD126/IL-6R |
| NM_010560 | Il6st | Interleukin 6 signal transducer | 5133400A03Rik/AA389424 |
| NM_009909 | Il8rb | Interleukin 8 receptor, beta | CD128/CDw128 |
| NM_008401 | Itgam | Integrin alpha M | CD11b/CD18 |
| NM_008404 | Itgb2 | Integrin beta 2 | 2E6/AI528527 |
| NM_010735 | Lta | Lymphotoxin A | LT/LT-[a] |
| NM_008518 | Ltb | Lymphotoxin B | AI662801/LTbeta |
| NM_010798 | Mif | Macrophage migration inhibitory factor | GIF/Glif |
| NM_007926 | Scye1 | Small inducible cytokine subfamily E, member 1 | EMAPII/Emap2 |
| NM_009263 | Spp1 | Secreted phosphoprotein 1 | AA960535/AI790405 |
| NM_011577 | Tgfb1 | Transforming growth factor, beta 1 | TGF-beta1/Tgfb |
| NM_013693 | Tnf | Tumor necrosis factor | DIF/TNF-alpha |
| NM_011609 | Tnfrsf1a | Tumor necrosis factor receptor superfamily, member 1a | CD120a/FPF |
| NM_011610 | Tnfrsf1b | Tumor necrosis factor receptor superfamily, member 1b | CD120b/TNF-R-II |
| NM_011616 | Cd40lg | CD40 ligand | CD154/Cd40l |
| NM_023764 | Tollip | Toll interacting protein | 4930403G24Rik/4931428G15Rik |
| NM_011798 | Xcr1 | Chemokine (C motif) receptor 1 | Ccxcr1/GPR5 |
| NM_010368 | Gusb | Glucuronidase, beta | AI747421/Gur |
| NM_013556 | Hprt1 | Hypoxanthine guanine phosphoribosyl transferase 1 | C81579/HPGRT |
| NM_008302 | Hspcb | Heat shock protein 1, beta | 90kDa/AL022974 |
| NM_001001303 | Gapdh | Glyceraldehyde-3-phosphate dehydrogenase | Gapd |
| NM_007393 | Actb | Actin, beta, cytoplasmic | Actx/E430023M04Rik |

**Table S1**. Genes analyzed in relation to inflammation.
